# Influence of Plasma Treatment on the Bonding Strength and Hydrophilicity of Zirconia: A Systematic Review and Meta-Analysis

**DOI:** 10.7759/cureus.105752

**Published:** 2026-03-24

**Authors:** Sahil Shaikh, Manish R Chauhan, Arti P Gangurde, Niraja Jaiswal, Radha L Harimkar, Sanjana Bhosale

**Affiliations:** 1 Prosthodontics and Crown and Bridge, Government Dental College and Hospital, Mumbai, IND

**Keywords:** adhesive dentistry, resin cement, surface modification, wettability, yttria-stabilized zirconia

## Abstract

Achieving reliable adhesion between resin-based materials and zirconia remains challenging because zirconia has a chemically inert surface and low intrinsic wettability. Plasma surface treatment has been proposed as a non-destructive method to improve surface energy, hydrophilicity, and bonding performance without altering the bulk structure of zirconia. However, variations in plasma type, gas composition, exposure duration, and testing protocols have limited clear conclusions regarding its effectiveness. This systematic review and meta-analysis were conducted in accordance with the Preferred Reporting Items for Systematic Reviews and Meta-Analyses (PRISMA) 2020 guidelines and registered in the International Prospective Register of Systematic Reviews (PROSPERO) (reference ID: CRD42025640563). Electronic searches of PubMed/MEDLINE, Scopus, Embase, Web of Science, and the Cochrane Library were performed up to January 31, 2026. Fourteen in vitro studies met the inclusion criteria. Risk of bias was assessed using the QUIN (Quality Assessment Tool for In Vitro Studies) tool. Overall, plasma-treated zirconia showed improved wettability and enhanced bond strength compared with untreated controls. Several studies also reported bond strength values comparable to or greater than those achieved with alumina sandblasting, particularly when plasma treatment was combined with 10-methacryloyloxydecyl dihydrogen phosphate (MDP)-containing primers. A meta-analysis showed a significant improvement in shear bond strength for plasma-treated zirconia compared with untreated zirconia (standardized mean difference (SMD) 3.04; 95% CI: 1.76-4.32), although heterogeneity was high. In the comparison with sandblasting, the primary pooled analysis was not significant, but the sensitivity analysis favored plasma treatment. Plasma surface treatment appears to be a promising strategy for improving zirconia bonding, although standardized protocols and clinical studies are still needed.

## Introduction and background

Zirconia has become a cornerstone material in restorative dentistry owing to its superior mechanical strength, biocompatibility, and excellent esthetics [1]. Compared to metal-based restorations, zirconia offers greater translucency and a tooth-like appearance, making it ideal for prosthetic rehabilitation. However, its inherently low surface energy and poor wettability present major challenges to reliable bonding with resin-based cements [2,3]. The lack of hydrophilicity hinders interaction with veneering ceramics and luting agents, ultimately compromising long-term clinical success. To address these limitations, several surface modification techniques, including mechanical, chemical, and plasma-based, have been proposed [4]. Among them, plasma treatment has gained attention as a non-invasive and effective approach to enhance surface characteristics without altering the bulk properties of zirconia [5].

Plasma treatment uses ionized gases to modify a material's surface at the molecular level, increasing its surface free energy and hydrophilicity while promoting chemical reactivity [6,7]. Common plasma delivery systems evaluated in dental materials research include cold atmospheric plasma (CAP) and atmospheric pressure plasma jet (APPJ) [6,7]. Depending on the gas used, such as oxygen, nitrogen, argon, or hydrogen, different reactive species are generated, influencing the formation of hydroxyl or nitrogen-containing groups that favor bonding with adhesive monomers [8-10]. The effectiveness of plasma treatment depends on variables such as exposure time, energy intensity, and gas composition, all of which determine its impact on bonding performance [11].

Unlike silica-based ceramics that can be etched with hydrofluoric acid, zirconia is resistant to conventional etching, necessitating alternative strategies like air abrasion or tribochemical silica coating [12-14]. However, these methods are technique-sensitive. When performed under recommended parameters, air abrasion can improve surface roughness and enhance micromechanical retention; nevertheless, inappropriate particle size, pressure, or exposure duration may still contribute to surface defects, undesirable surface degradation, and excessive tetragonal-to-monoclinic phase transformation [15]. Plasma treatment, by contrast, modifies zirconia at the atomic level, improving surface reactivity and supporting both micromechanical interlocking and chemical bonding without direct mechanical damage [7,16]. Laboratory-based plasma-assisted coating techniques such as plasma-enhanced chemical vapor deposition have also been explored, with Bitencourt et al. reporting improved zirconia-veneering ceramic bond strength without significantly altering surface roughness [17].

Enhanced hydrophilicity following plasma treatment facilitates improved adhesive wetting and interaction with phosphate monomers such as 10-methacryloyloxydecyl dihydrogen phosphate (MDP), thereby strengthening the chemical bond and enhancing the long-term durability of zirconia-resin interfaces [18,19]. From a clinical perspective, such improvements are meaningful only if they translate into more durable bonding after aging and more predictable cementation outcomes. Clinical evidence on ceramic veneer bonding highlights the importance of surface conditioning for predictable clinical success and should be considered when translating in vitro plasma findings to practice [20]. In addition, plasma-treated zirconia has demonstrated greater resistance to hydrothermal degradation while preserving its tetragonal crystalline phase, a key factor for maintaining mechanical stability under intraoral conditions [9]. Despite these promising outcomes, current evidence remains fragmented due to considerable variability in plasma sources, gas compositions, exposure durations, and energy settings. Moreover, much of the available research is limited to in vitro studies with inconsistent testing protocols, making it difficult to establish standardized recommendations for clinical application.

Given these inconsistencies and the absence of a consolidated evaluation of the literature, there is a clear need for a systematic review that critically assesses the effect of plasma surface treatment on the bonding strength and hydrophilicity of zirconia. Therefore, the present review aims to synthesize and appraise the available evidence to determine whether plasma treatment can reliably enhance adhesion between zirconia and resin-based cements, to identify gaps that warrant further investigation, and to help guide more standardized and clinically relevant future research in this area.

## Review

Methodology

The present systematic review and meta-analysis were designed and conducted in accordance with the Preferred Reporting Items for Systematic Reviews and Meta-Analyses (PRISMA) 2020 guidelines (checklist attached in Appendix A) [21]. The review protocol was prospectively registered in the International Prospective Register of Systematic Reviews (PROSPERO) under the reference ID CRD42025640563. The objective of this review was to evaluate and synthesize available evidence regarding the influence of plasma treatment on the bonding strength and hydrophilicity of zirconia used in dental applications.

Focused Question

The research question was formulated using the PICOS (Population, Intervention, Comparison, Outcome, Study design) framework. The population included zirconia-based dental materials utilized in restorative and prosthetic dentistry. The intervention of interest was plasma surface treatment of zirconia. The comparator consisted of untreated zirconia or zirconia subjected to other surface modification methods. The primary outcomes were bonding strength (such as shear bond strength, tensile bond strength, or microtensile bond strength) and hydrophilicity (evaluated through contact angle measurements or surface energy analysis). Eligible study designs included in vitro, in vivo, and clinical trials published in the English language with full text available. The focused research question was as follows: "What is the influence of plasma treatment on the bonding strength and hydrophilicity of zirconia in dental applications?".

Search Strategy

A comprehensive and systematic electronic search was performed across PubMed/MEDLINE, Scopus, Embase, Web of Science, and the Cochrane Library to identify relevant studies published from database inception to January 31, 2026. The search strategy combined controlled vocabulary and free-text terms related to zirconia, plasma treatment, bond strength, adhesion, and hydrophilicity. The core electronic search strategy was structured around the following concepts: (zirconia OR Y-TZP OR 3Y-TZP OR 3-YSZ OR yttria-stabilized zirconia) AND (plasma OR cold atmospheric plasma OR CAP OR atmospheric pressure plasma jet OR APPJ OR low-pressure plasma OR non-thermal plasma OR plasma-enhanced chemical vapor deposition OR PECVD) AND (bond strength OR shear bond strength OR tensile bond strength OR microshear OR microtensile OR adhesion OR resin cement) AND (hydrophilicity OR wettability OR contact angle OR surface energy). Search syntax was adapted for each database, and the complete database-specific search strategies are provided in Appendix B. The search was restricted to studies published in English, and only full-text articles were considered. Non-English articles were excluded because translation resources were not available for standardized full-text assessment. This language restriction was applied for feasibility and consistency of screening and data extraction; however, it may have excluded relevant studies published in other languages and therefore represents a potential source of language bias. No restrictions were applied regarding the year of publication or study design. To improve completeness, grey-literature searches were also performed in Google Scholar (first 300 records sorted by relevance), OpenGrey, and institutional repositories using combinations of the terms zirconia, plasma, bond strength, and hydrophilicity. Reference lists of all included studies were also manually screened to identify additional eligible articles.

Eligibility Criteria

Studies were included if they evaluated zirconia-based dental materials used in restorative or prosthetic applications and assessed the effects of plasma surface treatment on bond strength and/or hydrophilicity. Eligible designs included in vitro studies reporting quantitative outcomes related to the mechanical properties of the materials. Studies involving mixed ceramic materials were included only when zirconia-specific data could be extracted separately. Bond strength outcomes included shear bond strength, tensile bond strength, or microtensile/microshear bond strength, while hydrophilicity outcomes included contact angle, wettability, or surface energy measurements. Only studies with sufficiently described plasma treatment protocols and clearly reported outcome data were included. Exclusion criteria comprised studies unrelated to dental zirconia, studies on non-dental ceramics or non-zirconia substrates, case reports, narrative reviews, letters, editorials, and studies lacking quantitative data or a clearly reproducible plasma protocol. In addition, studies evaluating industrial or laboratory-scale plasma coating/deposition systems intended primarily for manufacturing workflows rather than direct chairside surface conditioning were excluded from the review.

Study Selection Process

Study selection was performed in two sequential stages by two independent reviewers (S.S. and M.C.). In the first stage, titles and abstracts were screened for relevance based on three core criteria: dental zirconia substrate, plasma-based surface treatment, and at least one quantitative outcome related to bond strength or hydrophilicity. In the second stage, the full texts of potentially eligible studies were retrieved and assessed against the predefined inclusion and exclusion criteria. Any disagreements between reviewers were resolved by discussion, and unresolved discrepancies were adjudicated by a third reviewer (A.G.).

Data Extraction

Data extraction was carried out using a pre-piloted extraction form to ensure uniformity. The extracted information included the author and year of publication, country, study design, sample characteristics, and type of zirconia used. Details of the intervention, such as plasma treatment type, gas composition, exposure duration, and power settings, were recorded. Outcome data for bonding strength (shear, tensile, or microtensile) and hydrophilicity (contact angle, wettability, surface energy) were also extracted. The comparator group, typically untreated or differently treated zirconia, and key findings, including statistical significance, were documented. Data were entered into a structured spreadsheet and independently verified by two reviewers for accuracy. The studies were subsequently categorized according to intervention type (e.g., atmospheric pressure plasma versus low-pressure plasma), zirconia variant, and evaluation method.

Risk of Bias Assessment

The methodological quality of in vitro studies was assessed using the QUIN (Quality Assessment Tool for In Vitro Studies) instrument [22]. This tool evaluates eight critical domains: clarity of objectives, sample size justification, control or randomization, blinding of assessors, adherence to standardized experimental protocols, validity of outcome measurement, appropriateness of statistical analysis, and conflict-of-interest disclosure. Each domain was rated as low, moderate, or high risk of bias. An overall risk category was assigned based on the number and severity of high-risk domains. Two reviewers (S.S. and M.C.) independently performed the assessment, and disagreements were resolved by discussion and consensus, with a third reviewer (A.G.) arbitration where required. This standardized approach facilitated the consistent evaluation of internal validity across diverse study designs.

Data Synthesis and Statistical Analysis

A qualitative narrative synthesis was first undertaken to summarize the effects of plasma treatment on zirconia surface properties and bonding outcomes. Quantitative synthesis was performed only when studies were sufficiently comparable with respect to substrate, comparator, and outcome reporting. For pooled analyses of shear bond strength, effect estimates were calculated as standardized mean differences (SMDs) with 95% confidence intervals (CIs) using Review Manager (RevMan 5.4) (The Cochrane Collaboration, London, England, United Kingdom) under an inverse-variance random-effects model. Separate meta-analyses were performed for plasma treatment versus untreated control and plasma treatment versus sandblasting. Statistical heterogeneity was assessed using the chi-squared (χ²) test and quantified using the I² statistic. Because methodological and clinical heterogeneity were anticipated, random-effects models were applied. To better explore between-study variability, studies were also grouped descriptively according to plasma modality, gas type, exposure duration, adjunctive MDP primer use, and aging interval before testing. Sensitivity analysis was performed by excluding studies that showed an outlying or directionally inconsistent effect on the pooled estimate. Funnel plot interpretation and Egger's regression test were not possible because of the limited number of pooled studies (fewer than 10).

Results

The present systematic review synthesized findings from multiple in vitro studies that evaluated the effects of plasma treatment on zirconia surfaces. Different plasma treatment conditions, gas compositions, exposure times, pressure settings, and comparator protocols were analyzed to determine their influence on bond strength and surface characteristics. The detailed characteristics and principal findings of the included studies are presented in Table 1.

**Table 1:** Data related to the characteristics and outcomes of the included studies Y-TZP: yttria-stabilized tetragonal zirconia polycrystal; 3Y-TZP: 3 mol% yttria-stabilized tetragonal zirconia polycrystal; 3-YSZ: 3 mol% yttria-stabilized zirconia; (Y,Nb)-TZP: yttria/niobium-stabilized tetragonal zirconia polycrystal; ZrO₂: zirconium dioxide; Y₂O₃: yttrium oxide; HF: hydrofluoric acid; PECVD: plasma-enhanced chemical vapor deposition; CAP: cold atmospheric plasma; APPJ: atmospheric pressure plasma jet; APDBD: atmospheric pressure dielectric barrier discharge; CAD/CAM: computer-aided design/computer-aided manufacturing; MDP: 10-methacryloyloxydecyl dihydrogen phosphate; SBS: shear bond strength; MPa: megapascal; CH₄: methane; HMDSO: hexamethyldisiloxane; Ar: argon; O₂: oxygen; N₂: nitrogen; CO₂: carbon dioxide; H₂: hydrogen; Al₂O₃: aluminum oxide (alumina)

Authors, year, study design	Zirconia material/type	Sample size	Plasma treatment type	Plasma pressure condition	Plasma temperature	Plasma treatment distance	Plasma exposure time	Plasma gas composition	Comparator(s)	Outcome measure (bond strength)	Outcome assessment method	Conclusion/key findings
Ito et al., 2016 [23], Japan, in vitro experimental study	Zirconia (Katana, Kuraray)	27 specimens (9 per group)	Atmospheric pressure low-temperature plasma treatment	Atmospheric (ambient)	Low temperature (value not provided)	10 mm	30 seconds	Helium	Control: untreated. Other: alumina sandblast treatment	Shear bond strength (MPa)	Shear adhesion tests using a universal testing machine (AGSJ-5kN, Shimadzu) at 0.5 mm/min after 24 h water immersion at 37°C	Plasma treatment enhances the bonding strength of adhesive resin cement to zirconia to a level equivalent to alumina sandblasting while preserving the zirconia crystal structure
Liu et al., 2016 [24], China, in vitro experimental study	Y-TZP plates (94% ZrO₂ stabilized by 5% Y₂O₃, Cercon Smart Ceramics)	30 Y-TZP plates (10 per group) + 10 lithium silicate plates (positive control)	Atmospheric cold plasma brush (non-thermal plasma)	Atmospheric (ambient)	Near room temperature (non-thermal)	5 mm	2 min (or 5 min for one group)	Argon (3 slm) and oxygen (30 sccm)	Control: negative control (untreated Y-TZP). Other: 2-min plasma; 5-min plasma; positive control (HF etching and silane on lithium silicate)	Shear bond strength (MPa)	Microshear test using a device on an Instron 3367 at 1 mm/min after 24 h storage at 37°C	Non-thermal plasma treatment significantly enhances the shear bond strength of Y-TZP, with 2‐min and 5‐min treatments increasing bond strength by 64.6% and 88.6%, respectively; 5‐min plasma achieved bond strength comparable to conventional HF etching/silane on lithium silicate
Tabari et al., 2017 [25], Iran, in vitro experimental study	Cercon® zirconia ceramics (base-colored, from DeguDent, Hanau, Germany)	180 discs (30 per group)	Non-thermal plasma treatment via a low-density cold active plasma beam	Atmospheric	Cold active (near room temperature)	10 mm	20 seconds	Varies by group: oxygen; argon; air; 20% O₂ + 80% Ar; 10% O₂ + 90% Ar	Control: untreated ceramics	Microshear bond strength (MPa)	Microshear test using a microtensile tester (Bisco) at 1 mm/min after light curing (40 s) and 24 h incubation at 37°C	Non-thermal plasma processing using various gas compositions decreases the contact angle (increases surface energy) and, particularly with air and 20% O₂ + 80% Ar, significantly enhances the microshear bond strength of Cercon® zirconia ceramics to resin composite cements
da Silva et al., 2018 [26], Brazil, in vitro experimental study	Y-TZP (VITA In-Ceram® YZ for inLab®)	48 specimens (n=16 per group)	Non-thermal oxygen plasma treatment using a hollow-cathode plasma gun	Base pressure of 2.0×10⁻⁴ mbar (prior to treatment)	Non-thermal (near room temperature)	120 mm	16 minutes	Oxygen	Control (untreated) and ceramic primer (Clearfil Ceramic Primer)	Microshear bond strength (MPa)	Microshear test using Tygon tube molds with Panavia V5 resin cement; tested after 24 h water storage at 37°C	Non-thermal oxygen plasma significantly increased the surface energy and microshear bond strength of zirconia compared to untreated and primer-treated groups, without altering surface roughness, suggesting it as an effective alternative surface treatment prior to cementation
Mahrous et al., 2018 [27], Egypt, in vitro experimental study	Zirconia plates fabricated from IPS e.max ZirCad blocks (Ivoclar Vivadent, Schaan, Liechtenstein)	48 plates (n=12 per group)	Non-thermal air atmospheric plasma treatment using Piezobrush® PZ2	Atmospheric	~50°C	5 mm	80 seconds	Air (atmospheric plasma)	Control (untreated); SD: 50 µm alumina sandblasting; PL: plasma treatment; SP: sandblasting + plasma treatment	Shear bond strength (MPa) of self-adhesive resin cement (Relay X Unicem, 3M ESPE)	Shear bond strength test using Instron Model 3345 at 0.5 mm/min after 8 days of water storage at 37°C	Non-thermal air plasma treatment significantly increases the shear bond strength of adhesive resin cement to zirconia, with the highest values obtained when plasma is combined with sandblasting. This protocol is a promising clinical approach for cementing zirconia restorations without compromising surface integrity
Yan et al., 2020 [28], Taiwan, in vitro experimental study	Zirconia (CAD/CAM zirconia; specific type not detailed in the abstract)	Not specified (assumed ~10 specimens per group)	Oxygen plasma treatment at varying powers (100, 200, and 400 W)	Working pressure set to 0.6 mbar after evacuation (<0.4 mbar)	Non-thermal (room temperature)	Not specified	Not specified (constant exposure time)	Oxygen	Control group: zirconia without plasma treatment (both pristine and sandblasted)	Shear bond strength (MPa)	Shear bond strength test using a computer-controlled material testing machine (e.g., Instron) at a defined crosshead speed	Oxygen plasma treatment of zirconia significantly improves the bond strength to composite resin without damaging the zirconia's microstructure, with higher plasma power yielding greater bond strength
Bunz et al., 2021 [29], Germany, in vitro experimental study	3-YSZ zirconia discs	12 specimens per group (6 groups)	CAP as an additional treatment	Argon gas at 2.5 bar; system stabilized for 5 min and then ambient pressure	Not explicitly specified	10 mm	60 seconds	Argon	A/SU: air abrasion + Scotchbond Universal; A/C: air abrasion + Clearfil Ceramic Primer; A/M: air abrasion + MKZ Primer; A/MP: air abrasion + Monobond Plus; S/SU: silica-coating + Scotchbond Universal; AP/SU: air abrasion + CAP + Scotchbond Universal	Shear bond strength (MPa)	Shear bond strength test after artificial aging (short term: 14 days/5000 thermal cycles; long term: 250 days/37,500 thermal cycles)	Although A/SU showed the highest bond strength in the short term, prolonged aging decreased shear bond strength in all groups except AP/SU, which exhibited the highest long-term durability. CAP treatment thus improves long-term adhesive durability between the veneering composite and zirconia
Yoda et al., 2022 [30], Japan, in vitro experimental study	3-YSZ (Aadva EI Zirconia Disc, GC, Tokyo, Japan)	60 specimens (5 per each of 12 experimental groups)	Low-temperature atmospheric pressure multi‑gas plasma jet treatment	Working pressure: not explicitly stated; ambient conditions maintained	20°C (room temperature)	3 mm above the specimen	3 s and 10 s (varied)	N₂; CO₂; O₂; Air; Ar	Untreated control group. Alumina sandblasting group (50 µm alumina; 5 s at 0.3 MPa from 5 mm)	Tensile bond strength between zirconia and resin cement	Tensile adhesion strength test using a universal testing machine (Autograph AG-I 20 kNT, Shimadzu) at 1.0 mm/min	Low‑temperature atmospheric pressure multi‑gas plasma treatment enhances zirconia surface wettability and reduces carbon contamination, thereby significantly improving resin cement-zirconia bond strength. The effect is dependent on the gas species and irradiation time, with N₂ plasma yielding the highest improvement
Ye et al., 2022 [31], Japan, in vitro experimental study	High‑translucency zirconia (VITA In‑Ceram® YZ for inLab®)	5 specimens per group; 6 groups (total n=30)	CAP jet treatment via a pen‑type APDBD plasma generator (CAP Med‑I)	Working pressure: backfilled to 0.6 mbar after evacuation (using high‑purity helium)	Not explicitly specified (assumed near room temperature)	10 mm	90 s	Helium (99.999%)	ZrT: untreated; ZrT‑M: MDP‑containing primer; ZrT‑A: air abrasion with 50 µm alumina; ZrT‑AM: air abrasion + MDP primer; ZrT‑P: plasma‑treated; ZrT‑PM: plasma‑treated + MDP primer	Shear bond strength (MPa)	Tensile adhesion strength test using a universal testing machine at 1 mm/min after 24 h water storage and thermal cycling	CAP treatment significantly enhanced the resin cement–zirconia bond strength, and its combination with an MDP‑containing primer (ZrT‑PM) produced the highest immediate and aged shear bond strength, suggesting a promising method for durable bonding of high‑translucency zirconia restorations
Salimi et al., 2023 [32], Iran, in vitro experimental study	3Y-TZP zirconia discs	40 specimens (10 per group; 4 groups)	Non-thermal argon plasma treatment	Atmospheric pressure	Near room temperature	10 mm	60 s	Argon	Control: untreated; sandblasting; plasma; plasma + MDP-containing primer	Shear bond strength (MPa)	Shear bond strength test using a universal testing machine at 1 mm/min after 24 h water storage	Non-thermal argon plasma treatment significantly enhanced the resin cement–zirconia bond strength compared to the untreated control, with the combination of plasma and MDP-containing primer yielding the highest bond strength, indicating its potential for durable clinical application
Alalawi et al., 2024 [33], Saudi Arabia, in vitro experimental study	Y-TZP zirconia discs; lithium disilicate cylinders (CAD-on assembly)	70 specimens of lithium disilicate; 70 specimens of Y-TZP zirconia	APPJ treatment via Piezobrush® PZ3 (piezoelectric direct discharge)	Ambient conditions using device settings (power set at 80%; pressure details provided with sandblasting as 2 bar for that treatment)	Assumed near room temperature	5 mm	30 s	Ambient air (device operates with ambient air)	(C) Control: no treatment. (S) Sandblasting only (50 µm Al₂O₃, 10 s, 15 mm, 2 bar). (P) Plasma only. (SP) Sandblasting + plasma. (SN) Sandblasting + universal adhesive. (PN) Plasma + universal adhesive. (SPN) Sandblasting + plasma + universal adhesive	Shear bond strength (MPa)	Shear bond strength test using a universal testing machine at 1 mm/min (after water storage and cementation)	APPJ treatment significantly reduced the contact angle (improved wettability) without altering surface topography; its shear bond strength was comparable to sandblasting. However, adding universal adhesive reduced bond strength. APPJ is a promising, non-destructive surface treatment method for CAD-on ceramic assemblies
Jiang et al., 2024 [34], China, in vitro experimental study	Y-TZP zirconia discs	40 specimens (10 per group; 4 groups)	CAP treatment	Atmospheric pressure	Room temperature	10 mm	60 s	Argon	Control: untreated; sandblasting (50 µm Al₂O₃); plasma; plasma + MDP-containing primer	Shear bond strength (MPa)	Shear bond strength test using a universal testing machine at 1 mm/min after 24 h water storage	CAP treatment significantly improved zirconia's bond strength to resin cement. Plasma combined with an MDP-containing primer yielded the highest shear bond strength, making it a promising approach for durable clinical applications
Kim et al., 2024 [35], South Korea, in vitro experimental study	3Y-TZP (Vatech MCIS, Korea); (Y,Nb)-TZP (Vatech MCIS, Korea)	60 specimens (5 per each of 12 experimental groups)	Low-temperature atmospheric pressure forming gas plasma treatment	Atmospheric (ambient)	Room temperature	3 mm above the specimen	2 seconds	5% H₂ in N₂	Control (polished). Sandblasting. Plasma. Sandblasting + plasma	Shear bond strength (MPa)	Universal testing machine at 1 mm/min after 24 h water storage	Plasma treatment alone resulted in a significant decrease in bond strength, but when combined with sandblasting, it significantly improved bond strength in (Y,Nb)-TZP specimens. Sandblasting increased bond strength in both groups, but (Y,Nb)-TZP exhibited higher values with plasma treatment
Miura et al., 2024 [36], Japan, Finland, in vitro experimental study	Prettau zirconia (3Y-TZP, Zirkonzahn)	192 specimens (24 per group; 8 groups)	Low-temperature atmospheric pressure plasma treatment	Atmospheric pressure	Room temperature	5 mm	30 s	Air or argon	Control (alumina blasting only). Air plasma (P). Argon plasma (AP). Katana cleaner (K). Ozonated water (OZ). OZ + AP. AP + K. Tap water + AP (WAP)	Shear bond strength (MPa)	Universal testing machine at 1 mm/min after 24 h water storage	Argon plasma significantly improved zirconia's bond strength, with the highest values observed when combined with Katana Cleaner (AP + K) and ozonated water (OZ + AP). Plasma-treated surfaces showed enhanced wettability, supporting their use for long-term durable bonding in zirconia restorations

Study Characteristics

**Figure 1:**
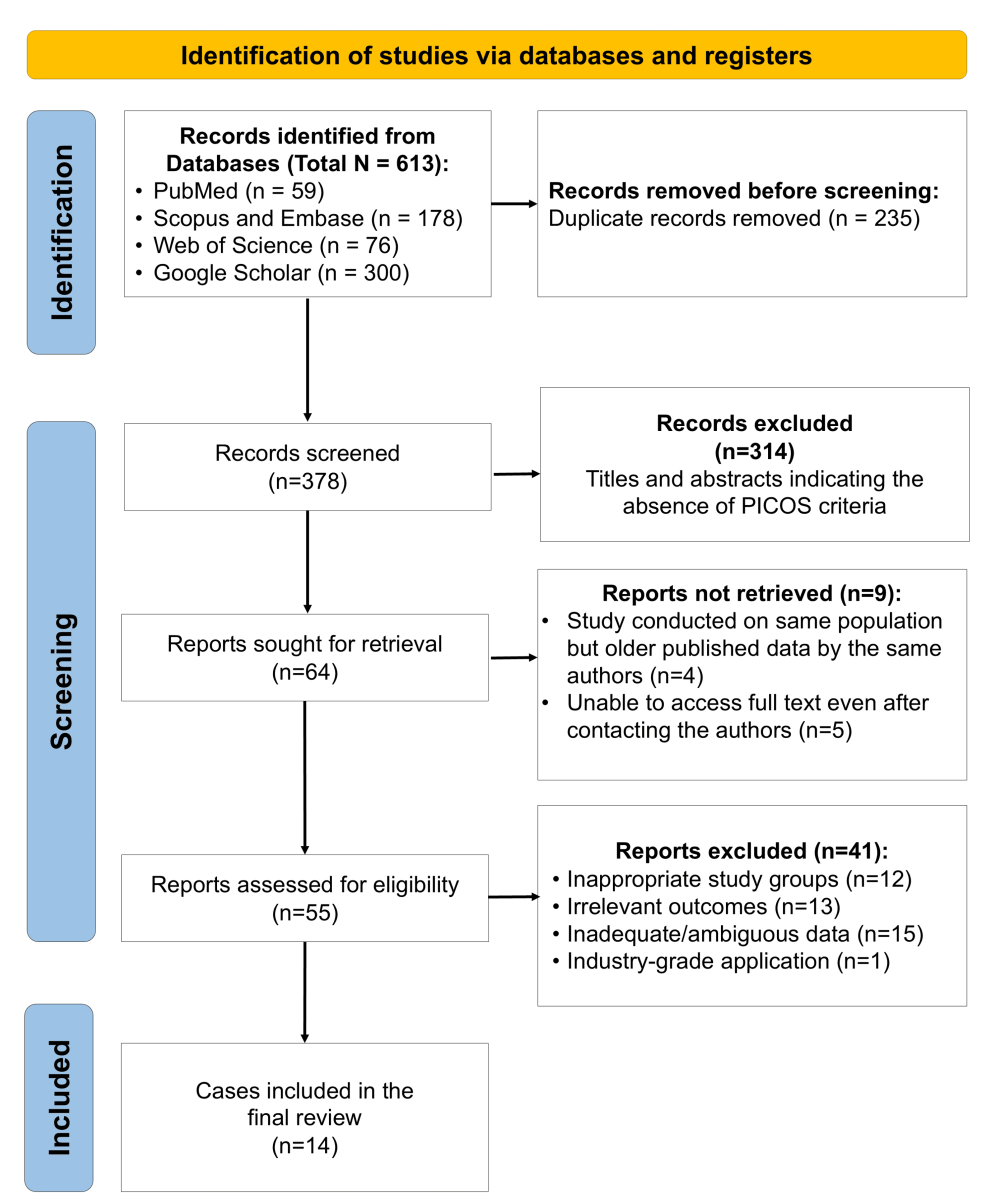
PRISMA flow diagram indicating the selection process of the articles in the present systematic review PRISMA: Preferred Reporting Items for Systematic Reviews and Meta-Analyses; PICOS: Population, Intervention, Comparison, Outcome, Study design

A total of 14 studies were included in this review (Figure 1). These studies were published between 2016 and 2024 and originated from multiple countries, including Japan, China, Iran, Brazil, Egypt, Taiwan, Germany, Saudi Arabia, South Korea, and Finland [23-36]. All included investigations were in vitro experimental studies evaluating the effect of plasma treatment on zirconia used in dental applications. The zirconia materials assessed included conventional yttria-stabilized tetragonal zirconia polycrystal (Y-TZP), 3 mol% yttria-stabilized tetragonal zirconia polycrystal (3Y-TZP), high-translucency zirconia, and computer-aided design/computer-aided manufacturing (CAD/CAM) zirconia. Sample sizes varied considerably across studies, ranging from small multi-group experimental designs to larger specimen-based studies. Most studies compared plasma treatment with untreated zirconia, while several also included comparisons with alumina sandblasting, MDP-containing primers, or combined conditioning protocols.

Plasma Treatment Parameters

The included studies investigated a broad spectrum of plasma treatment modalities. CAP and other non-thermal atmospheric plasma systems were the most commonly studied approaches [23-36]. In addition, APPJ, oxygen plasma, argon plasma, multi-gas plasma jet systems, and forming gas plasma were also evaluated. Most studies employed atmospheric pressure or ambient condition plasma systems, while a smaller number used low-pressure conditions or pre-evacuation steps before treatment [26,28,31].

The majority of studies described plasma application as being performed at room temperature or under non-thermal conditions, emphasizing the preservation of the zirconia substrate while improving its surface reactivity [23-36]. Only Mahrous et al. reported an approximate treatment temperature of 50°C [27]. Plasma application distance ranged from 3 mm to 120 mm, although most studies used distances between 5 mm and 10 mm [23-36]. Exposure times varied markedly from two seconds to 16 minutes, with many studies using short treatment durations between 30 seconds and 90 seconds [23-36]. A variety of gases were used, most commonly argon and oxygen, followed by helium, air, nitrogen, and mixed-gas systems such as 20% O₂ + 80% Ar and 5% H₂ in N₂ [23-36].

Bond Strength Measurements and Comparators

Most included studies evaluated shear bond strength, while two studies assessed tensile bond strength between zirconia and resin cement [30,31]. Universal testing machines were used across all studies for bond strength assessment. In most investigations, testing was conducted after 24-hour water storage, whereas some studies additionally included thermocycling, short-term aging, or prolonged artificial aging to assess bond durability over time [29,31]. All studies included an untreated zirconia control group. The most common comparator was alumina sandblasting, followed by MDP-containing primers [23,27,29-36]. Some studies also evaluated combined treatment approaches, such as plasma plus sandblasting, plasma plus universal adhesive, plasma plus MDP primer, or plasma in combination with auxiliary surface-cleaning agents such as Katana Cleaner and ozonated water [27,31-36]. One study also compared plasma-treated zirconia with hydrofluoric acid etching and silane-treated lithium silicate, serving as a positive control for ceramic bonding [24].

Synthesis of Results

Across the included studies, untreated zirconia consistently exhibited lower bond strength values, confirming the limited intrinsic adhesion of zirconia to resin-based materials without surface modification. Overall, plasma-treated zirconia demonstrated improved wettability and enhanced bond strength in comparison with untreated controls [23-36]. These improvements were attributed mainly to increased surface energy, reduced contact angle, enhanced surface cleanliness, and improved interaction with adhesive monomers. Several studies demonstrated that plasma treatment produced bond strength values that were comparable to or greater than those achieved by conventional alumina sandblasting [23,24,30,33,34,36]. Ito et al. reported that atmospheric pressure low-temperature plasma treatment enhanced zirconia-resin bond strength to a level comparable with alumina sandblasting while preserving the zirconia crystal structure [23]. Liu et al. showed that non-thermal plasma treatment significantly improved the microshear bond strength of Y-TZP, with longer exposure producing greater enhancement [24]. Yoda et al. similarly found that multi-gas atmospheric plasma treatment improved resin cement-zirconia bond strength, with the magnitude of improvement depending on gas type and irradiation time [30].

Several studies also highlighted the beneficial role of combined treatment approaches. Mahrous et al. observed that plasma treatment significantly increased zirconia bond strength, with the highest values seen when plasma was combined with sandblasting [27]. Ye et al. and Jiang et al. found that plasma treatment used together with an MDP-containing primer produced the highest immediate and/or aged bond strength values, suggesting a synergistic effect between plasma-induced surface activation and chemical adhesive bonding [31,34]. Salimi et al. also reported superior bond strength when argon plasma was combined with an MDP-containing primer compared with untreated or singly treated surfaces [32]. Long-term performance was specifically addressed in the study by Bunz et al., who reported that although some conventional treatment groups showed higher short-term bond strength, prolonged aging reduced bond strength in all groups except the plasma-treated group combined with air abrasion and universal adhesive, which demonstrated the highest long-term durability [29]. This finding suggests that plasma treatment may contribute not only to immediate bond improvement but also to better maintenance of adhesion over time in selected protocols.

Not all studies showed uniformly positive results for plasma used alone. Kim et al. reported that plasma treatment by itself reduced bond strength in some groups, whereas its combination with sandblasting significantly improved bonding in yttria/niobium-stabilized tetragonal zirconia polycrystal ((Y,Nb)-TZP) zirconia [35]. This finding indicates that the effectiveness of plasma treatment may depend on the zirconia composition, gas mixture, and whether it is used alone or as an adjunct to other conditioning methods. Alalawi et al. similarly found that APPJ treatment significantly improved wettability and produced bond strength comparable to sandblasting, although the addition of universal adhesive reduced bond strength in their CAD-on ceramic assembly model [33]. Miura et al. reported that argon plasma significantly improved zirconia bond strength, particularly when used together with Katana Cleaner or ozonated water, suggesting that plasma may also work favorably with adjunctive surface decontamination strategies [36]. Collectively, these findings indicate that plasma treatment is a promising method for improving zirconia bonding, although the magnitude of benefit varies according to plasma modality, gas composition, treatment duration, comparator protocol, and the use of adjunctive primers or cleaning agents.

Risk of Bias

Among the included studies, most were judged to have a low overall risk of bias, while a smaller number were rated as having moderate risk of bias (Table 2). Low-risk studies generally demonstrated clearly stated objectives, appropriate control groups, use of standardized experimental protocols, valid outcome assessment methods, and suitable statistical analyses. The studies rated as moderate risk mainly showed limitations related to incomplete reporting of sample size justification, blinding, group allocation, or methodological standardization. Importantly, no study was classified as having a high overall risk of bias. Overall, the predominance of low-risk studies supports the methodological reliability of the evidence included in this review.

**Table 2:** Risk of bias across the included in vitro studies using the QUIN tool QUIN: Quality Assessment Tool for In Vitro Studies

Study identification	Objective clarity	Sample size justification	Randomization/control	Blinding of assessors	Standardized protocols	Outcome assessment validity	Statistical analysis appropriateness	Conflict of interest	Overall risk of bias
Ito et al., 2016 [23]	Low	Low	Low	Low	Low	Low	Low	Low	Low
Liu et al., 2016 [24]	Low	Low	Moderate	Moderate	Low	Moderate	Low	Low	Moderate
Tabari et al., 2017 [25]	Low	Low	Moderate	Moderate	Low	Low	Moderate	Low	Moderate
da Silva et al., 2018 [26]	Low	Low	Low	Low	Low	Low	Low	Low	Low
Mahrous et al., 2018 [27]	Low	Low	Moderate	Low	Low	Low	Low	Low	Moderate
Yan et al., 2020 [28]	Low	Low	Low	Low	Low	Low	Low	Low	Low
Bunz et al., 2021 [29]	Low	Low	Moderate	Low	Low	Low	Moderate	Low	Moderate
Yoda et al., 2022 [30]	Low	Low	Low	Moderate	Low	Low	Low	Low	Low
Ye et al., 2022 [31]	Low	Low	Low	Low	Low	Low	Low	Low	Low
Salimi et al., 2023 [32]	Low	Low	Low	Low	Low	Low	Low	Low	Low
Alalawi et al., 2024 [33]	Low	Low	Low	Moderate	Low	Low	Low	Low	Moderate
Jiang et al., 2024 [34]	Low	Low	Moderate	Low	Low	Low	Low	Low	Moderate
Kim et al., 2024 [35]	Low	Low	Low	Low	Low	Low	Low	Low	Low
Miura et al., 2024 [36]	Low	Low	Low	Low	Low	Low	Low	Low	Low

Meta-Analysis

**Figure 2:**
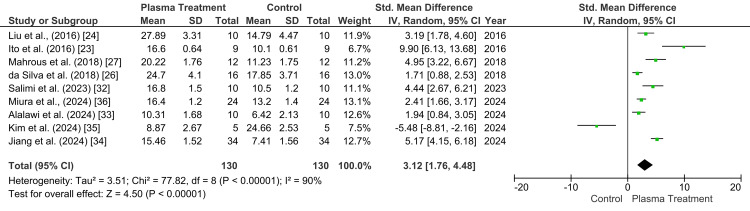
Forest plot showing the shear bond strength in control versus plasma treatment

The meta-analysis evaluating the effect of plasma treatment versus untreated control on the shear bond strength of zirconia included nine studies, with a total of 130 specimens in the plasma treatment group and 130 specimens in the control group (Figure 2). A random-effects model was used because substantial methodological heterogeneity was present among the included studies. The pooled SMD was 3.04 (95% CI: 1.76 to 4.32), demonstrating a statistically significant improvement in shear bond strength following plasma treatment (p<0.00001). However, heterogeneity was considerable (p<0.00001; I²=89%), indicating marked variability across the included studies. Despite this heterogeneity, the direction of effect favored plasma treatment in the majority of studies, supporting its beneficial role in improving zirconia bond strength relative to untreated surfaces.

**Figure 3:**
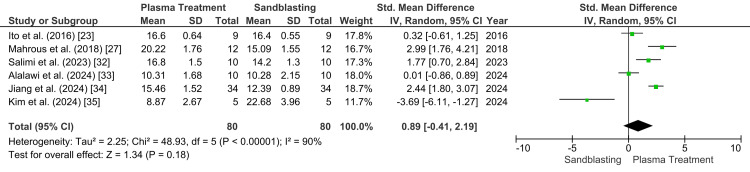
Forest plot showing the shear bond strength after sandblasting versus plasma treatment

The meta-analysis comparing plasma treatment versus sandblasting for zirconia shear bond strength initially included six studies with 80 specimens in each group (Figure 3). Using a random-effects model, the pooled SMD was 0.89 (95% CI: −0.41 to 2.19), indicating no statistically significant overall difference between plasma treatment and sandblasting (p=0.18). Heterogeneity was considerable (p<0.00001; I²=90%), suggesting marked variability across the included studies. Most studies favored plasma treatment, but Kim et al. showed a strong effect in the opposite direction, contributing substantially to the inconsistency in the pooled estimate [35].

**Figure 4:**
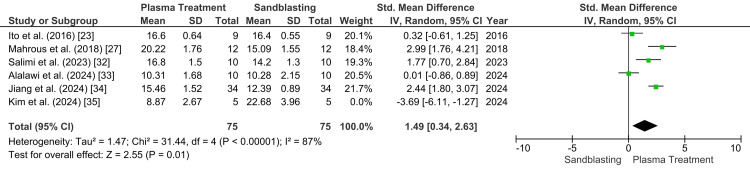
Forest plot after performing a sensitivity analysis by removing the outliers showing the advantage of plasma treatment over sandblasting in terms of shear bond strength

A subsequent sensitivity analysis was therefore performed after the exclusion of Kim et al. [35]. Following this adjustment, the pooled effect shifted in favor of plasma treatment, with an SMD of 1.49 (95% CI: 0.34 to 2.63), demonstrating a statistically significant advantage of plasma treatment over sandblasting (p=0.01). Although heterogeneity remained high (p<0.00001; I²=87%), the sensitivity analysis suggests that the overall conclusion was influenced by one directionally discordant study (Figure 4). Study weights were assigned using the inverse-variance random-effects method and were not equal, with individual contributions ranging from 16% to 18.8% in the primary analysis and 18.4% to 21.7% after sensitivity adjustment. Overall, these findings suggest that plasma treatment may provide superior bond strength compared with sandblasting under selected conditions, although the pooled estimate should still be interpreted cautiously because of persistent heterogeneity.

Discussion

Adhesion of resin-based materials to zirconia continues to be a major challenge in restorative dentistry because zirconia combines excellent mechanical properties with a relatively inert and low-energy surface that does not readily bond to resin cements [37-40]. Unlike silica-based ceramics, zirconia does not respond predictably to conventional hydrofluoric acid etching and silanization protocols, which limits the effectiveness of routine ceramic conditioning methods [3,12,40]. This has led to continued interest in alternative surface modification strategies that can improve zirconia bonding without compromising its structural integrity. In this context, plasma treatment has emerged as a promising non-abrasive approach because it can modify only the outermost surface layer while preserving the bulk properties of the material [6,8,9].

The principal mechanism behind plasma treatment appears to be physicochemical surface activation rather than macroscopic roughening. Exposure to plasma generates reactive species that reduce surface carbon contamination, increase surface free energy, and improve wettability, thereby creating a more favorable substrate for resin infiltration and adhesive interaction [9,11,18,19]. This is particularly relevant for phosphate-containing adhesive systems, since improved wetting may facilitate more effective interaction between zirconia and functional monomers such as MDP [41,42]. The findings of the present review support this mechanism, as most included studies reported a reduction in contact angle together with improved bond strength after plasma treatment [23-36].

The included studies consistently showed that plasma treatment enhanced bonding performance when compared with untreated zirconia, although the magnitude of improvement varied across protocols. Ito et al. demonstrated that atmospheric pressure low-temperature helium plasma improved zirconia-resin bond strength to a level comparable to alumina sandblasting while preserving the zirconia crystal structure [23]. Liu et al. similarly reported that argon-oxygen plasma significantly increased bond strength, with longer exposure producing greater improvement [24]. Tabari et al. found that non-thermal plasma treatment reduced contact angle and increased microshear bond strength, with air and oxygen-argon mixtures showing particularly favorable results [25]. da Silva et al. also reported that oxygen plasma increased both surface energy and microshear bond strength without altering surface roughness, suggesting that plasma may enhance chemical receptivity even in the absence of marked topographic change [26].

The influence of plasma treatment, however, was not uniform and clearly depended on protocol-related factors such as gas composition, treatment time, and energy delivery. Mahrous et al. found that short-duration atmospheric air plasma significantly improved bond strength, with the highest values obtained when plasma was combined with sandblasting [27]. Yan et al. observed that higher oxygen plasma power was associated with greater bond strength, indicating that energy intensity may be an important determinant of treatment efficacy [28]. Yoda et al. reported that the bonding effect of atmospheric multi-gas plasma varied according to gas species and irradiation time, with nitrogen plasma showing the most favorable results in their model [30]. Ye et al. further demonstrated that helium-based CAP improved both immediate and aged bond strength, especially when combined with an MDP-containing primer, highlighting the value of combining plasma activation with chemical adhesion strategies [31].

A recurring finding across several studies was that plasma performed particularly well when used as an adjunct rather than as a fully isolated treatment. Bunz et al. reported that while conventional groups showed favorable short-term bond strength, the plasma-associated group demonstrated the best long-term durability after prolonged aging, suggesting that plasma may contribute to better maintenance of adhesion over time [29]. Salimi et al. and Jiang et al. both found that argon-based plasma combined with an MDP-containing primer yielded the highest bond strength values, indicating a possible synergistic effect between improved surface activation and phosphate-monomer bonding [32,34]. Miura et al. also reported favorable bond enhancement with argon plasma, particularly when combined with auxiliary cleaning approaches such as Katana Cleaner and ozonated water [36]. These findings suggest that plasma may be best considered as part of a multimodal zirconia conditioning strategy rather than as a complete replacement for all existing protocols.

At the same time, the reviewed studies also make it clear that plasma treatment cannot be regarded as universally superior under all conditions. Kim et al. observed that plasma treatment alone reduced bond strength in some experimental groups, whereas the combination of sandblasting and plasma improved bonding in yttria/niobium-stabilized zirconia [35]. Alalawi et al. reported that APPJ treatment improved wettability and produced bond strength comparable to sandblasting, but not clearly superior to it; they also found that the addition of universal adhesive reduced bond strength in their CAD-on assembly model [33]. These findings are important because they show that plasma effectiveness depends not only on the plasma source itself but also on the zirconia type, associated adhesive system, and whether the surface is pretreated mechanically or chemically. Thus, the present evidence supports plasma as a promising conditioning method, but not as a one-size-fits-all solution.

When compared with conventional zirconia conditioning methods, plasma offers several practical and biological advantages. Air abrasion and related mechanical surface treatments remain clinically useful and may enhance micromechanical retention when properly performed, but they are also technique-sensitive and have been associated with surface damage, phase transformation, and changes in mechanical behavior under certain conditions [15,38,39]. Plasma, in contrast, acts through surface activation rather than abrasive alteration and therefore offers a dry, non-destructive, and chairside-applicable method for improving adhesive behavior [6,8,9]. The present review suggests that plasma can achieve bond strength comparable to sandblasting in many situations and may even outperform it in selected protocols, especially when combined with MDP-containing primers or other adjunctive conditioning steps [23,27,31-36].

The quantitative synthesis in this review also supports the potential benefit of plasma treatment, but it must be interpreted with caution. Meta-analysis was feasible for selected shear bond strength comparisons; however, heterogeneity remained high because of differences in plasma devices, gas composition, exposure duration, comparator protocols, aging methods, and zirconia type. In the comparison of plasma treatment versus sandblasting, the primary pooled analysis did not show a statistically significant difference, whereas the sensitivity analysis shifted the estimate in favor of plasma after the exclusion of one directionally discordant study. This indicates that the pooled outcome was influenced by protocol-level variation and that current evidence is still not sufficiently standardized to support a single universal clinical recommendation. Therefore, the meta-analytic findings should be viewed as supportive rather than definitive.

A limitation of the present review is that only English-language studies were included, which may have introduced language bias and led to the exclusion of relevant evidence published in other languages. Another important consideration is clinical translation. Most of the currently available evidence is derived from in vitro studies, and although these studies are essential for mechanistic understanding, they cannot fully reproduce the thermal, mechanical, chemical, and operator-related variables present in the oral environment. Clinical evidence on ceramic bonding also indicates that successful long-term outcomes depend not only on initial bond strength but on the stability of the entire adhesive protocol over time [20]. Clinical studies of bonding protocols in other dental applications demonstrate that laboratory gains do not always translate directly to clinical success, underscoring the need for clinical validation of plasma protocols [43]. For this reason, improvements seen in laboratory bond strength should not be assumed to translate directly into equivalent clinical success without further validation. Future research should therefore focus on protocol standardization, aging methods that better simulate clinical service, and well-designed clinical studies evaluating the long-term performance of plasma-conditioned zirconia restorations.

Overall, the present review indicates that plasma treatment is a promising and minimally invasive approach for improving zirconia surface wettability and resin bonding. The direction of evidence was largely favorable, and most included studies showed low risk of bias, which strengthens confidence in the general trend of findings. Nevertheless, substantial heterogeneity persists across plasma systems and testing methodologies. Future work should define the most effective gas compositions, exposure times, and adjunctive adhesive strategies so that plasma treatment can be translated into a more predictable and standardized clinical protocol for zirconia cementation.

## Conclusions

Plasma surface treatment appears to be a promising approach for improving the wettability and bonding performance of zirconia without causing evident structural damage to the material. The available in vitro evidence suggests that plasma-induced surface activation can enhance zirconia-resin adhesion and, in several protocols, may provide results comparable to or better than conventional surface treatments. However, the current evidence base remains heterogeneous with respect to plasma type, gas composition, exposure duration, and testing methods. Therefore, further standardization of treatment protocols and well-designed long-term clinical studies are necessary before plasma treatment can be recommended for routine clinical use in zirconia adhesive procedures.

## Appendices

Appendix A

**Table 3:** PRISMA checklist for the confirmation of the presence of each item in the present systematic review PRISMA: Preferred Reporting Items for Systematic Reviews and Meta-Analyses; IMRAD: Introduction, Methods, Results, and Discussion; PROSPERO: International Prospective Register of Systematic Reviews; PICOS: Population, Intervention, Comparison, Outcome, Study design

Section/topic		Checklist item	Yes/no
Title			
Title	1	Identify the report as a systematic review, meta-analysis, or both	Yes
Abstract			
Structured summary	2	Provide a structured summary (IMRAD) including, as applicable, Introduction (objectives); Methods (study eligibility criteria, participants, and interventions; study appraisal and synthesis methods); Results; and Discussion (limitations, conclusions, and implications of key findings) (systematic review registration number (PROSPERO))	Yes
Introduction			
Rationale	3	Describe the rationale for the review in the context of what is already known	Yes
Objectives	4	Provide an explicit statement of questions being addressed with reference to PICOS	Yes
Methods			
Protocol and registration	5a	Indicate if a review protocol exists and if and where it can be accessed (e.g., web address), and, if available, provide the registration information, including registration number	Yes
	5b	Registration on PROSPERO (preferable)	Yes
Eligibility criteria	6	Specify the study characteristics (e.g., PICOS, length of follow-up) and report characteristics (e.g., years considered, language, publication status) used as criteria for eligibility, giving a rationale	Yes
Information sources	7	Describe all information sources (e.g., databases with dates of coverage, contact with study authors to identify additional studies) in the search and date last searched	Yes
Search	8	Present a full electronic search strategy for at least one database, including any limits used, such that it could be repeated	Yes
Study selection	9	State the process for selecting studies (i.e., screening, eligibility-inclusion/exclusion criteria, included in the systematic review, and, if applicable, included in the meta-analysis)	Yes
Data collection process	10	Describe the method of data extraction from reports (e.g., piloted forms, independently, in duplicate) and any processes for obtaining and confirming data from investigators	Yes
Data items	11	List and define all variables for which data were sought (e.g., PICOS, funding sources) and any assumptions and simplifications made	Yes
Risk of bias in individual studies	12	Describe the methods used for assessing risk of bias of individual studies (including specification of whether this was done at the study or outcome level) and how this information is to be used in any data synthesis	Yes
Summary measures	13	State the principal summary measures (e.g., risk ratio, difference in means)	Yes
Synthesis of results	14	Describe the methods of handling data and combining results of studies, if done, including measures of consistency (e.g., I2) for each meta-analysis (only for meta-analysis study)	Yes
Risk of bias across studies	15	Specify any assessment of risk of bias that may affect the cumulative evidence (e.g., publication bias, selective reporting within studies)	Yes
Additional analyses	16	Describe the methods of additional analyses (e.g., sensitivity or subgroup analyses, meta-regression), if done, indicating which were pre-specified (only for meta-analysis study)	Yes
Results			
Study selection	17	Give the numbers of studies screened, assessed for eligibility, and included in the review, with reasons for exclusions at each stage, ideally with a flow diagram	Yes
Study characteristics	18	For each study, present the characteristics for which data were extracted (e.g., study size, PICOS, follow-up period) and provide the citations	Yes
Risk of bias within studies	19	Present the data on the risk of bias of each study and, if available, any outcome-level assessment (see item 12) (only for meta-analysis study)	Yes
Results of individual studies	20	For all outcomes considered (benefits or harms), present, for each study, (a) simple summary data for each intervention group and (b) effect estimates and confidence intervals, ideally with a forest plot (only for meta-analysis study)	Yes
Synthesis of results	21	Present the results of each meta-analysis done, including confidence intervals and measures of consistency (only for meta-analysis study)	Yes
Risk of bias across studies	22	Present the results of any assessment of risk of bias across studies (see item 15) (only if meta-analysis was performed)	Yes
Additional analysis	23	Give the results of additional analyses, if done (e.g., sensitivity or subgroup analyses, meta-regression (see item 16)) (only for meta-analysis study)	Yes
Discussion			
Summary of evidence	24a	Summarize the main findings including the strength of evidence for each main outcome; consider their relevance to key groups (e.g., healthcare providers, users, and policy makers)	Yes
	24b	Report the conflicting findings (from literature) and put forth new ideas and/or new research directions	Yes
Limitations	25	Discuss the limitations at the study and outcome levels (e.g., risk of bias) and at the review level (e.g., incomplete retrieval of identified research, reporting bias)	Yes
Conclusions	26	Provide a general interpretation of the results in the context of other evidence and implications for future research	Yes
Citations	27	Cite from recent literature in the articles	Yes
Funding			
Funding	28	Describe the sources of funding for the systematic review and other support (e.g., supply of data), the role of funders for the systematic review, and the grant number	Yes

Appendix B

**Table 4:** Full search strategies across each database

Database/source	Search date	Search strategy/terms used	Limits/notes
PubMed/MEDLINE	January 31, 2026	(("Zirconium Oxide"[Mesh] OR zirconia[tiab] OR "zirconium oxide"[tiab] OR Y-TZP[tiab] OR 3Y-TZP[tiab] OR "3-YSZ"[tiab] OR "yttria-stabilized zirconia"[tiab] OR "high-translucency zirconia"[tiab] OR "CAD/CAM zirconia"[tiab]) AND ("Plasma Gases"[Mesh] OR plasma[tiab] OR "cold atmospheric plasma"[tiab] OR CAP[tiab] OR "atmospheric pressure plasma jet"[tiab] OR APPJ[tiab] OR "low-pressure plasma"[tiab] OR "non-thermal plasma"[tiab] OR "atmospheric plasma"[tiab] OR "oxygen plasma"[tiab] OR "argon plasma"[tiab] OR "nitrogen plasma"[tiab] OR "helium plasma"[tiab] OR "forming gas plasma"[tiab] OR PECVD[tiab] OR "plasma-enhanced chemical vapor deposition"[tiab]) AND ("Tensile Strength"[Mesh] OR "Shear Strength"[Mesh] OR "Adhesiveness"[Mesh] OR "Dental Bonding"[Mesh] OR "bond strength"[tiab] OR adhesion[tiab] OR adhesive[tiab] OR "shear bond strength"[tiab] OR "tensile bond strength"[tiab] OR "microshear bond strength"[tiab] OR "microtensile bond strength"[tiab] OR luting[tiab] OR "resin cement"[tiab]) AND ("Surface Properties"[Mesh] OR hydrophilicity[tiab] OR hydrophilic[tiab] OR wettability[tiab] OR "contact angle"[tiab] OR "surface energy"[tiab] OR "surface free energy"[tiab]))	English language; full-text articles considered; no year restriction
Scopus	January 31, 2026	TITLE-ABS-KEY ((zirconia OR "zirconium oxide" OR "yttria-stabilized zirconia" OR Y-TZP OR 3Y-TZP OR "3-YSZ" OR "high-translucency zirconia" OR "CAD/CAM zirconia") AND (plasma OR "cold atmospheric plasma" OR CAP OR "atmospheric pressure plasma jet" OR APPJ OR "low-pressure plasma" OR "non-thermal plasma" OR "atmospheric plasma" OR "oxygen plasma" OR "argon plasma" OR "nitrogen plasma" OR "helium plasma" OR "forming gas plasma" OR PECVD OR "plasma-enhanced chemical vapor deposition") AND ("bond strength" OR adhesion OR adhesive OR "shear bond strength" OR "tensile bond strength" OR "microshear bond strength" OR "microtensile bond strength" OR "resin cement" OR luting) AND (hydrophilicity OR hydrophilic OR wettability OR "contact angle" OR "surface energy" OR "surface free energy"))	Limited to the English language; no year restriction
Embase	January 31, 2026	('zirconia'/exp OR zirconia:ti,ab OR 'zirconium oxide':ti,ab OR 'yttria stabilized zirconia':ti,ab OR y-tzp:ti,ab OR 3y-tzp:ti,ab OR '3-ysz':ti,ab OR 'high translucency zirconia':ti,ab OR 'cad/cam zirconia':ti,ab) AND ('plasma'/exp OR plasma:ti,ab OR 'cold atmospheric plasma':ti,ab OR cap:ti,ab OR 'atmospheric pressure plasma jet':ti,ab OR appj:ti,ab OR 'low pressure plasma':ti,ab OR 'non thermal plasma':ti,ab OR 'atmospheric plasma':ti,ab OR 'oxygen plasma':ti,ab OR 'argon plasma':ti,ab OR 'nitrogen plasma':ti,ab OR 'helium plasma':ti,ab OR 'forming gas plasma':ti,ab OR pecvd:ti,ab OR 'plasma enhanced chemical vapor deposition':ti,ab) AND ('bond strength':ti,ab OR adhesion:ti,ab OR adhesive:ti,ab OR 'shear bond strength':ti,ab OR 'tensile bond strength':ti,ab OR 'microshear bond strength':ti,ab OR 'microtensile bond strength':ti,ab OR 'resin cement':ti,ab OR luting:ti,ab) AND ('hydrophilicity':ti,ab OR hydrophilic:ti,ab OR wettability:ti,ab OR 'contact angle':ti,ab OR 'surface energy':ti,ab OR 'surface free energy':ti,ab)	English language; no year restriction
Web of Science Core Collection	January 31, 2026	TS=((zirconia OR "zirconium oxide" OR "yttria-stabilized zirconia" OR Y-TZP OR 3Y-TZP OR "3-YSZ" OR "high-translucency zirconia" OR "CAD/CAM zirconia") AND (plasma OR "cold atmospheric plasma" OR CAP OR "atmospheric pressure plasma jet" OR APPJ OR "low-pressure plasma" OR "non-thermal plasma" OR "atmospheric plasma" OR "oxygen plasma" OR "argon plasma" OR "nitrogen plasma" OR "helium plasma" OR "forming gas plasma" OR PECVD OR "plasma-enhanced chemical vapor deposition") AND ("bond strength" OR adhesion OR adhesive OR "shear bond strength" OR "tensile bond strength" OR "microshear bond strength" OR "microtensile bond strength" OR "resin cement" OR luting) AND (hydrophilicity OR hydrophilic OR wettability OR "contact angle" OR "surface energy" OR "surface free energy"))	Refined to the English language; no year restriction
Cochrane Library	January 31, 2026	((zirconia OR "zirconium oxide" OR "yttria-stabilized zirconia" OR Y-TZP OR 3Y-TZP OR "3-YSZ" OR "high-translucency zirconia" OR "CAD/CAM zirconia"):ti,ab,kw) AND ((plasma OR "cold atmospheric plasma" OR CAP OR "atmospheric pressure plasma jet" OR APPJ OR "low-pressure plasma" OR "non-thermal plasma" OR "atmospheric plasma" OR "oxygen plasma" OR "argon plasma" OR "nitrogen plasma" OR "helium plasma" OR "forming gas plasma" OR PECVD OR "plasma-enhanced chemical vapor deposition"):ti,ab,kw) AND (("bond strength" OR adhesion OR adhesive OR "shear bond strength" OR "tensile bond strength" OR "microshear bond strength" OR "microtensile bond strength" OR "resin cement" OR luting):ti,ab,kw) AND ((hydrophilicity OR hydrophilic OR wettability OR "contact angle" OR "surface energy" OR "surface free energy"):ti,ab,kw)	English language; no year restriction
Google Scholar	January 31, 2026	Search combinations used: "zirconia" plasma "bond strength"; "zirconia" plasma hydrophilicity; "zirconia" "cold atmospheric plasma"; "zirconia" APPJ bond strength; "zirconia" plasma "contact angle"	First 300 results screened by relevance
OpenGrey	January 31, 2026	Search combinations used: zirconia plasma bond strength; zirconia plasma hydrophilicity; zirconia cold atmospheric plasma; zirconia atmospheric pressure plasma jet; zirconia plasma contact angle	Grey literature searched for theses, dissertations, and conference materials where available
Institutional repositories	January 31, 2026	Search combinations used: zirconia plasma bond strength; zirconia plasma hydrophilicity; zirconia cold atmospheric plasma; zirconia atmospheric pressure plasma jet; zirconia plasma contact angle	Used to identify additional unpublished or difficult-to-locate academic material
Manual hand-searching	Up to January 31, 2026	Reference lists of all included studies and relevant review articles were manually screened for additional eligible studies	Performed after database screening
